# Comprehensive portrait of recurrent glioblastoma multiforme in molecular and clinical characteristics

**DOI:** 10.18632/oncotarget.5038

**Published:** 2015-09-04

**Authors:** Rui Li, Xincheng Chen, Yongping You, Xiefeng Wang, Yanwei Liu, Qi Hu, Wei Yan

**Affiliations:** ^1^ Department of Neurosurgery, The First Affiliated Hospital of Nanjing Medical University, Nanjing, China; ^2^ Beijing Neurosurgical Institute, Capital Medical University, Beijing, China

**Keywords:** recurrent glioblastoma, primary glioblastoma, molecular characteristics, clinical features

## Abstract

Glioblastoma multiforme is the most common primary malignant brain tumor in adults. In addition to poor response to treatment, a high recurrence rate contributes to the poor prognosis. The purpose of this study was to investigate the genetical and clinical characteristics of recurrent glioblastoma. We used whole transcriptome sequencing data to examine the distribution of molecular subtypes and gene signatures in 22 recurrent glioblastoma taken from the Chinese population, and further analyzed biological progression of the tumors, when compared with primary glioblastoma. The proportion of the classical subtype in recurrent ones (22%) was lower than that in primary glioblastoma (36%). The frequency of IDH1 mutations in recurrent glioblastomas was nearly twice that in primary glioblastomas. TP53 mutations were fewer in proneural recurrent glioblastomas (20%) but frequent in classical recurrent glioblastomas (80%). The most common sites of recurrent glioblastomas were the temporal lobe (41%). In patients diagnosed with recurrent glioblastoma multiforme, 64% were younger than 50 years. Gene set enrichment analysis revealed that chromatin fracture, repair, and remodeling genes were enriched in recurrent glioblastoma. Our results highlight the differences in clinical features, molecular subtypes and gene alterations between primary and recurrent glioblastoma and may be helpful for targeted therapy for recurrent glioblastoma.

## INTRODUCTION

Glioblastoma multiforme (GBM) is the most common and malignant primary brain tumor in adults. Despite advancing treatments including resection, radiotherapy and chemotherapy, most patients survive less than one year [1–4]. Numerous patient-specific factors result in a poor response to treatment, including the tumor acquiring resistance to treatment, tumor heterogeneity, and restricted delivery of treatments to the central nervous system as a consequence of both the blood–brain barrier and the high interstitial peritumoral pressures [5, 6]. The high recurrence rate found in patients with GBM is a major clinical challenge. Previous clinical trials have evaluated combination therapy for the treatment of recurrent GBM, and these are better than monotherapies [7–15]. Despite some minor improvements in progression-free survival (PFS) of recurrent GBM by the introduction of temozolomide (TMZ) and other targeted treatments, no satisfactory improvement in overall survival has been achieved [16]. In addition to a high rate of recurrence, the appearance of new genetic mutations and malignant phenotypes in the process of recurrence increases the difficulty of treatment for recurrent GBM [17, 18]. A comprehensive portrait of the genetic alterations, age distribution, tumor localization and other clinical features in primary and recurrent GBM is needed to better classify tumor profiles. This could lead to an understanding of the characteristics of recurrent GBM, and suggest potential targets for personalized therapeutic strategies.

In the present study, we investigated the characteristics of primary and recurrent GBM, with the goal of developing targeted and individualized treatment for GBM. We used whole transcriptome sequencing to assess 110 samples, including 88 primary GBM and 22 recurrent GBM, with defined molecular subtypes (proneural, neural, classical and mesenchymal). We analyzed the distribution of genetic events and examined the clinical features between the subtypes. We found that primary and recurrent GBM showed differences in mutation point distribution and clinical features. Gene set enrichment analysis (GSEA) indicated that the two types of GBM were enriched in different gene sets. Our findings provide a comprehensive portrait of gene alterations, clinical features and gene set enrichment in primary and recurrent GBM, which could be helpful in determining the direction of potential targeted drug therapy.

## RESULTS

### Distribution of molecular subtypes in primary and recurrent GBM

**Figure 1:**
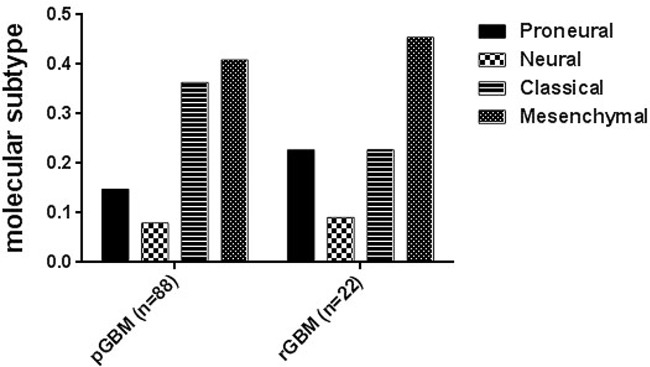
Distribution of molecular subtypes (Proneural, Neural, Classical and Mesenchymal) in primary and recurrent GBM Molecular subtypes are indicated in different bars.

As shown in Figure 1, 36% of 88 primary GBM belonged to the classical subtype. This was higher than the proportion of this subtype in recurrent GBM (22%). However, the proportion of the proneural subtype in primary GBM (15%) was lower than in recurrent GBM (23%). Samples identified as being of the neural subtype made up 8% of primary GBM and 9% of recurrent GBM. Approximately half of primary and recurrent GBM were identified as the mesenchymal subtype (41% and 45%, respectively).

### Investigation of gene alterations in primary and recurrent GBM

IDH1 mutations were found in 12 out of 88 primary GBM (14%) and 6 of 22 recurrent GBM (27%). The frequency of ATRX mutations in primary GBM was 10%, and 18% in recurrent GBM. TP53 mutation exhibited a slightly higher frequence in (59%) recurrent GBM than that of primary GBM (50%). EGFR mutations were detected in 26% of primary GBM, and in 18% of recurrent GBM. (Table 1)

**Table 1:** Gene alterations in primary and recurrent GBM according to their molecular subtypes

	Primary glioblastoma				Recurrent glioblastoma			
	Proneural	Neural	Classical	Mesenchymal	Proneural	Neural	Classical	Mesenchymal
No. of patients	13	7	32	36	5	2	5	10
IDH 1 mutation	10	0	2	0	4	0	1	1
TP53 mutation	5	5	14	20	1	1	4	7
EGFR mutation	4	1	8	10	2	0	1	1
ATRX mutation	2	2	2	3	0	0	1	3

Among proneural subtypes, both primary and recurrent GBM showed a high frequency of IDH1 mutations. TP53 mutations were detected in 5 of 13 proneural primary GBM but in only 1 out of 5 proneural recurrent GBM. Such mutations were also present in 14 of 32 (44%) classical primary GBM, but were even more prevalent in classical recurrent GBM (80%).

In the mesenchymal subtype, TP53, EGFR and ATRX mutations were detected in 20 (56%), 10 (28%) and 3 (8%) out of 36 mesenchymal primary GBM respectively, and 70%, 10% and 30% of 10 mesenchymal recurrent glioblastomas.

### Clinical feature of primary and recurrent GBM

**Figure 2:**
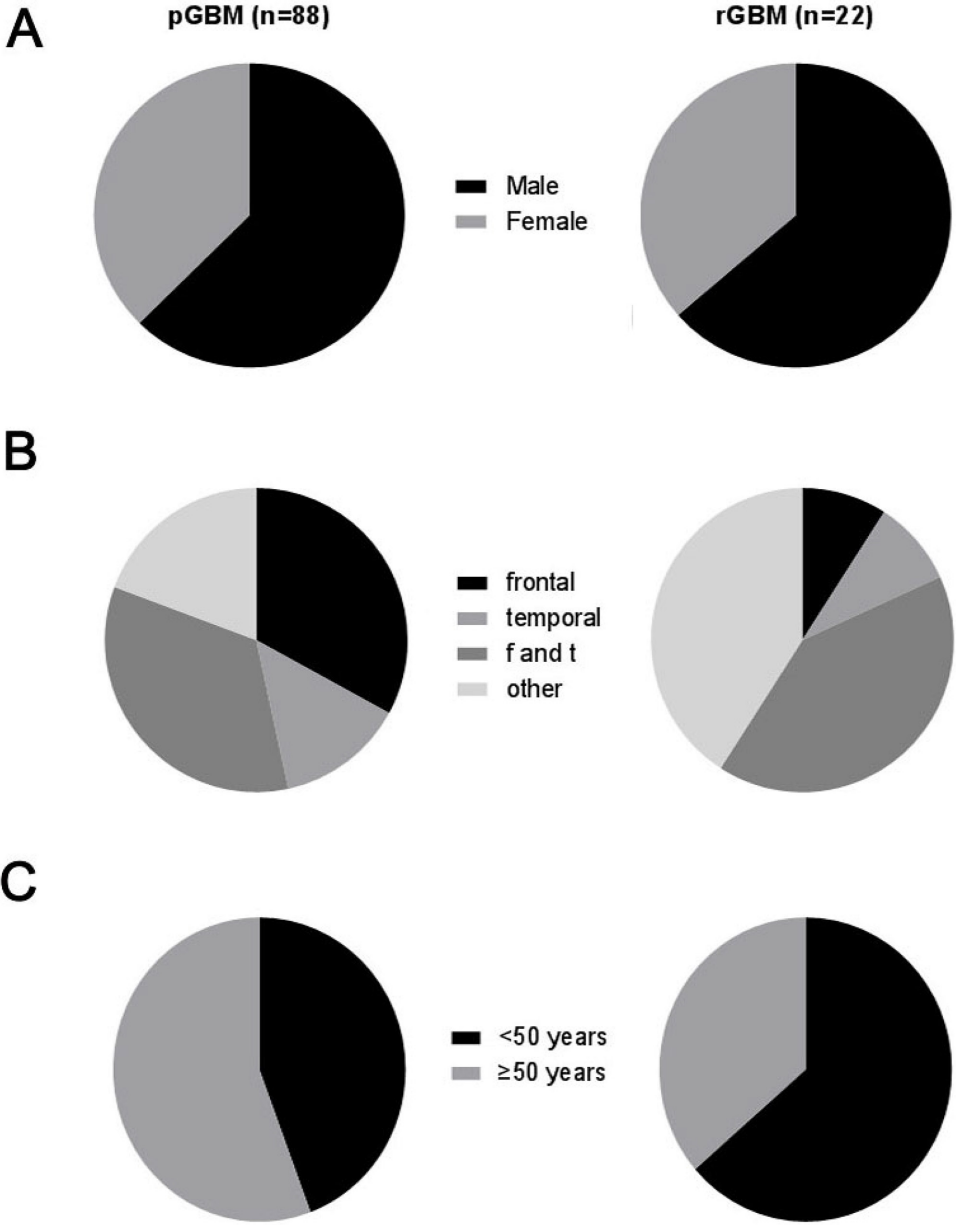
Clinical features of primary and recurrent GBM **A.** Distribution of gender with molecular subtypes of GBM; **B.** Distribution of anatomic localization with molecular subtypes of GBM (f and t: co-involving the frontal and temporal lobes. other lobes: excluding the frontal and temporal lobes); **C.** Age cohort of patients with four molecular subtypes of GBM.

GBM predominantly affected males; the male to female ratio was 1.67 in primary GBM and 1.75 in recurrent GBM (Figure 2A and Table 2). With respect to anatomic localization, the frontal and temporal lobes were the most common sites of primary GBM, accounting for 33% and 34%, respectively (Figure 2B). However, only 9% of recurrent GBM were located in the frontal lobe, which was significantly lower than that of recurrent GBM (*P* < 0.05). The most common sites of recurrent GBM were the temporal lobe (41%) and other lobes (excluding the frontal and temporal lobes, 41%, Figure 2C). Among the 88 patients with primary GBM, 39 patients were younger than 50 years of age, and 49 patients were 50 years of age or older (Figure 2B). Among the 22 patients with recurrent GBM, 14 patients were younger than 50 years and 8 patients were 50 years of age or older (Figure 2C). The mean age of patients diagnosed with primary GBM was 49.61 ± 1.346 years, and with recurrent GBM, the mean age was 47.73 ± 1.782 years.

**Table 2:** Clinical features of patients with primary and recurrent GBM

No. of patients	Age (years)		Gender		anatomical location			
	< 50	≥ 50	Male	Female	Frontal lobe	Temporal lobe	Both lobes*	Other lobes
Primary GBM	39	49	55	33	29	30	12	17
percentage	44%	56%	63%	37%	33%	34%	14%	19%
Recurrent GBM	14	8	14	8	2	9	2	9
percentage	64%	36%	64%	36%	9%	41%	9%	41%

* Both lobes: co-involved frontal and temporal lobes.

### Gene set enrichment analysis of primary and recurrent GBM

**Figure 3:**
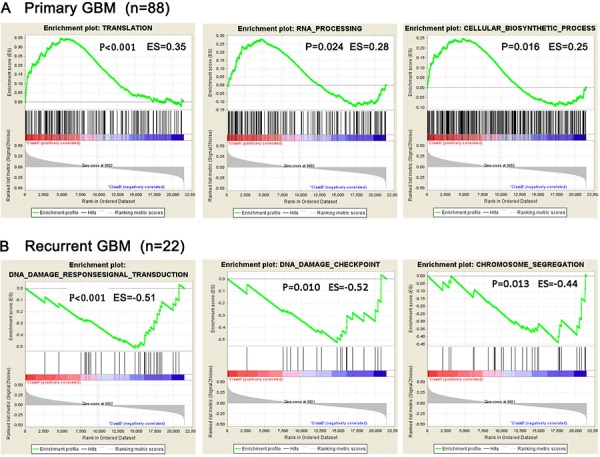
Presence of representative gene sets related to biological processes analyzed by GSEA **A.** Gene sets related to biological processes in 88 pGBM; **B.** Gene sets related to biological processes in 22 rGBM.

We performed GSEA on the whole transcriptome sequencing of 88 primary GBM and 22 recurrent GBM. Our results indicate that gene sets related to TRANSLATION (*P* < 0.001), RNA_PROCESSING (*P* = 0.024) and CELLULAR_BIOSYNTHETIC_PROCESS (*P* = 0.016) were significantly enriched in the primary GBM, while DNA_DAMAGE_RESPONSESIGNAL_TRANSDUCTION (*P* < 0.001), DNA_DAMAGE_CHECKPOINT (P = 0.010) and CHROMOSOME_SEGREGATION (P = 0.013) gene sets were enriched in recurrent GBM (Figure 3 and Supplementary Table S1).

## DISCUSSION

Glioblastoma multiforme (GBM) is the most common and aggressive malignant primary brain tumor in adults. Despite treatment involving chemotherapy, radiation and surgery, GBM exhibits a median survival rate of 15 months [3, 4, 19]. An important factor in the poor prognosis of primary GBM is a high recurrence rate [20]. Currently, treatments for recurrent GBM mainly involve repeated resection, further chemotherapy and focal irradiation [21, 22]. With the accumulation of knowledge regarding the molecular and genetic profile of GBM, some molecularly targeted therapies, such as growth factor inhibitors or TMZ, have led to some progress in treatment [3, 16]. However, despite these advances in treatment strategies, the majority of patients suffered recurrent GBM, with a subsequent median survival of approximately 6 months [23–26]. Therefore, insight into the differences between primary and recurrent glioblastoma from a molecular and clinical perspective, and exploration of more effective molecular targets for therapy is urgently. To date, the distinction between primary and recurrent GBM has not been systematically investigated. In the present study, 88 primary and 22 recurrent GBM were investigated using whole transcriptome sequencing combined with analysis of clinical data. When the 110 tumors were divided into molecular subtypes, we found that the proportion of proneural subtype was more frequent in recurrent GBM than that of primary GBM, while the classical subtype showed an opposite result. The incidence of neural and mesenchymal subtypes between primary and recurrent GBM remained similar. These results suggest that the proneural subtype was more likely to recurrent when compared to the other molecular subtypes. The classical subtype exhibited the opposite pattern of incidence.

The frequency of IDH1 mutations in recurrent GBM was nearly twice that of primary GBM, and distribution of this group of mutations was particularly high in the proneural subtype. TP53 mutations were more likely to be found in classical and mesenchymal recurrent GBM than in primary GBM. However, such mutations were rare in proneural recurrent GBM. The phenomena of variable distribution of molecular subtypes and gene signatures between primary and recurrent GBM suggests that these genetic events might play important roles in the recurrent progression of primary GBM. For example, classical and mesenchymal primary GBM exhibiting TP53 mutation had a high risk of recurrence, whereas proneural primary GBM carrying TP53 mutation were less likely to relapse.

The ratio of male to female incidence of primary and recurrent GBM was approximately the same, demonstrating that there was no difference in recurrence of GBM depending on gender. With respect to anatomic localization, 33% of primary GBM were located in frontal lobe, whereas only 9% of recurrent GBM were found there. Conversely, other lobes, especially temporal lobe, were more common sites for recurrent GBM than primary GBM. These findings suggest that tumors in frontal lobe had a low recurrent rate. There was no significant difference in mean age between primary and recurrent GBM. However, when we used 50-year-old as a boundary line to divided into 2 groups, less than 50 and more than or equal to 50, we found that 64% of patients with recurrent GBM were younger than 50 years old, compared with 44% of primary GBM. Because there is no difference in age between primary and recurrent GBM in the whole cohort, we cannot conclude that people younger than 50 years old have a higher tendency towards recurrence. However, the results above suggest that the recurrence rate of primary GBM does not increase with age.

Aimed for finding the enriched gene sets among the differentially expressed genes between primary and recurrent tumors, GSEA was performed for primary and recurrent GBM in this study. Enriched gene sets related to TRANSLATION, RNA_PROCESSING and CELLULAR_BIOSYNTHETIC_PROCESS were found in primary GBM. These are related to protection and progression of primary GBM, while DNA_DAMAGE_RESPONSESIGNAL_TRANSDUCTION, DNA_DAMAGE_CHECKPOINT and CHROMOSOME_SEGREGATION gene sets relating to recurrence of tumor cells were enriched in recurrent GBM. Compared to primary GBM, recurrent tumors displayed significant biological progression, including DNA damage repair, cell metabolic process and other rebuilt procession of recurrent tumor cells. This was consistent with the clinical progression of recurrent GBM relapsed from the residual cancer cells after treatments.

Our study comprehensively characterizes the distinction in molecular subtypes, gene signatures and clinical features between primary and recurrent GBM. Although past efforts have not significantly improved the prognosis of recurrent GBM, increasing insight into primary and recurrent GBM can help us to enhance timely intervention and reduce the recurrence rate of GBM, as well as improving molecularly targeted therapies.

## MATERIALS AND METHODS

### Tumor samples

A total of 110 glioblastoma multiforme (GBM) samples from the Chinese Glioma Genome Atlas were included in this study, consisting of 88 primary GBM (pGBM) and 22 recurrent GBM (rGBM). Tumor tissue samples were obtained by surgical resection. All pGBM and rGBM cases were classified by two neuropathologists according to the 2007 WHO classification guidelines and Scherer [27]. Only samples with greater than 80% tumor cells were selected. All patients provided written informed consent, and the study was approved by the ethics committees of the participating hospitals.

### Whole transcriptome sequencing

Whole transcriptome sequencing was performed as described previously [28]. Briefly, total RNA was isolated from homogenized frozen tissue samples using the RNeasy Mini Kit (Qiagen) according to the manufacturer's instructions. RNA purity was checked using a 2100 Bioanalyzer (Agilent Technologies) and only high quality samples with an RNA Integrity Number (RIN) value greater than or equal to 7.0 were used to construct the sequencing library. The subsequent sequencing steps included end repair, adapter ligation, size selection and polymerase chain reaction enrichment. The length of DNA fragment was measured using a 2100 Bioanalyzer, with a median insert size of 200 nucleotides. The libraries were sequenced on the Illumina HiSeq 2000 platform using the 101-bp pair-end sequencing strategy. Short sequence reads were aligned to the human reference genome (Hg 19 Refseq) using the Burrows-Wheeler Aligner (BWA, Version 0.6.2-r126). SnpEff software was used to annotate genetic variance [29].

### Gene set enrichment analysis

To determine the gene sets related to particular biological processes present in pGBM and rGBM, gene expression profiling and gene set enrichment analysis (GSEA) was performed as described previously [30].

### Statistical analysis

Student's *t*-test was performed using SPSS 13.0. All data are presented as the mean ± SEM. A *P*-value of < 0.05 was considered significant.

## SUPPLEMENTARY MATERIALS TABLE


