# Supplementary material for: Comprehensive portrait of recurrent glioblastoma multiforme in molecular and clinical characteristics
Source: Oncotarget. 2015 Sep 4;6(31):30968–74. doi: 10.18632/oncotarget.5038 (PMC4741581; doi:10.18632/oncotarget.5038)
Supplement: Supplementary file 1 [file oncotarget-06-30968-s001.pdf]

SUPPLEMENTARY TABLE

Supplementary Table S1: Gene sets (TOP 20) enriched in recurrent GBM

	NAME	ES	NES	NOM *p*-val
1	DNA_DAMAGE_RESPONSESIGNAL_TRANSDUCTION	–0.51	–1.89	0.000
2	MICROTUBULE_CYTOSKELETON_ORGANIZATION_AND_BIOGENESIS	–0.50	–1.84	0.000
3	HETEROCYCLE_METABOLIC_PROCESS	–0.52	–1.79	0.004
4	REGULATION_OF_MUSCLE_CONTRACTION	–0.57	–1.78	0.006
5	REGULATION_OF_CELL_ADHESION	–0.48	–1.76	0.006
6	LOCOMOTORY_BEHAVIOR	–0.38	–1.75	0.000
7	BEHAVIOR	–0.34	–1.68	0.000
8	DNA_DAMAGE_CHECKPOINT	–0.52	–1.67	0.010
9	SULFUR_COMPOUND_BIOSYNTHETIC_PROCESS	–0.54	–1.64	0.026
10	DNA_INTEGRITY_CHECKPOINT	–0.48	–1.60	0.018
11	ENDOSOME_TRANSPORT	–0.49	–1.59	0.025
12	ESTABLISHMENT_AND_OR_MAINTENANCE_OF_CELL_POLARITY	–0.51	–1.59	0.024
13	REGULATION_OF_ENDOCYTOSIS	–0.52	–1.59	0.014
14	MICROTUBULE_BASED_PROCESS	–0.36	–1.59	0.004
15	CHROMOSOME_SEGREGATION	–0.44	–1.58	0.013
16	CELLULAR_PROTEIN_CATABOLIC_PROCESS	–0.38	–1.56	0.010
17	ENERGY_RESERVE_METABOLIC_PROCESS	–0.53	–1.56	0.042
18	RESPONSE_TO_ORGANIC_SUBSTANCE	–0.44	–1.55	0.021
19	REGULATION_OF_DNA_METABOLIC_PROCESS	–0.39	–1.54	0.006
20	POTASSIUM_ION_TRANSPORT	–0.37	–1.52	0.021
